# First record of the adventive oriental aphid *Schizaphis piricola* (Matsumura, 1917) (Hemiptera, Aphididae) in Europe

**DOI:** 10.3897/zookeys.89.903

**Published:** 2011-04-11

**Authors:** Pérez Hidalgo Nicolás, Umaran Ángel, Mier Durante M. Pilar

**Affiliations:** 1 *Departamento de Biodiversidad y Gestión Ambiental, Universidad de León. 24071. León, España*; 2 *Asociación “Fotografía y Biodiversidad”, Torrejón de Ardoz. 28850 Madrid, España*

**Keywords:** *Schizaphis piricola*, aphids, adventive species, Spain

## Abstract

The oriental aphid Schizaphis piricola (Matsumura) is recorded for the first time in Europe, on the ornamental pear tree Pyrus calleryana in landscaped areas in Madrid (Spain). Data on the morphology of the forms on primary host (apterous and alate fundatrigeniae and fundatrices), and their biology and distribution are given. The keys for identifying species of Schizaphis (Schizaphis) in the Iberian Peninsula are updated. Two species of aphids are also recorded for the first time on Pyrus calleryana: Schizaphis piricola and Aphis pomi.

## Introduction

**Figure 1.**
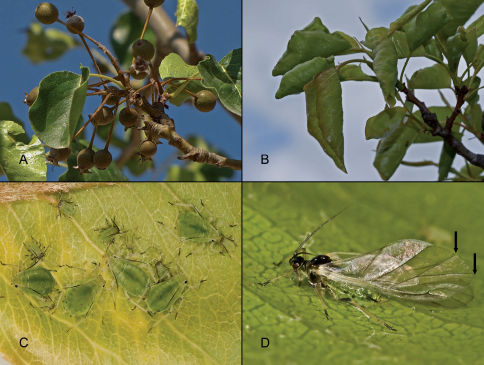
Pyrus calleryana with fruit (**A**), malformation caused by Schizaphis piricola (Matsumura) on Pyrus calleryana leaves (**B**), apterous fundatrigeniae (**C**) and alate fundatrigeniae (**D**) of Schizaphis piricola, the arrows indicate the tips of the two branches of the medial vein.

The genus Schizaphis Börner contains approximately 36 Palearctic species and 6 Nearctic ones. Is a genus resembling Rhopalosiphum Koch with little differences between both genus, and for this reason require further taxonomical and molecular study (Blackman and Eastop 1994; Foottit et al. 2008). The species of Schizaphis are characterised by more or less cylindrical siphunculi slightly constrained at the apex, ultimate rostral segment short and heart size, absence of dorsal cuticular ornamentation, and alatae with two branches on the medial vein of the wings (Fig. 1D) (Pérez Hidalgo and Mier Durante 2005). Most of the species in the genus are monoecious on species of Poaceae, Juncaceae and Cyperaceae but a few mainly oriental species, are dioecious with species of Pyrus as the primary host, where they lay their cold-resistant eggs (Blackman and Eastop 2006).

Twenty-seven species have been recorded in Europe (Holman 2009; Nieto Nafría et al. 2010): 21 in the nominal subgenus, 5 in the subgenus Paraschizaphis and one in the subgenus Euschizaphis. Eight of them were recorded in the Iberian Peninsula (Pérez Hidalgo and Mier Durante 2005; Blackman and Eastop 2006): Schizaphis graminum (Rondani), Schizaphis longicaudata Hille Ris Lambers, Schizaphis pyri Shaposhnikov, Schizaphis rotundiventris (Signoret), Schizaphis (Euschizaphis) palustris (Theobald), Schizaphis (Paraschizaphis) caricis (Schouteden), Schizaphis (Paraschizaphis) rosazevedoi (Ilharco) and Schizaphis (Paraschizaphis) scirpi (Passerini). Of these, only Schizaphis pyri, has Pyrus communis as its primary host and Cyperaceae as its secondary host; the rest live on Poaceae or Cyperaceae without causing financial loss, except for Schizaphis graminum which can be a cereal pest (Blackman and Eastop 2000).

A photograph of a colony of aphids on the pear tree of oriental origin Pyrus calleryana Decne in “Juan Carlos I park”, Barajas (Madrid, Spain) (Fig. 1) taken on 26th April, 2009 and posted on the “Biodiversidad Virtual” portal (http://www.biodiversidadvirtual.org/) enabled the oriental species Schizaphis piricola (Matsumura) to be detected for the first time in Europe. Its presence was confirmed in a study of samples collected the following spring on the same host and in the same place. Its route of entry into Europe is probably linked to when the host plant was imported, as is the case of many other species introduced into Europe (Coeur d’acier et al. 2010).

This finding is yet another example of how social networks play an important role in our knowledge of biodiversity and the detection and/or monitoring of invasive or endangered species (Pérez Hidalgo et al. 2009; Silverton 2010).

## Studied material

Samples containing several apterae (3 fundatrices and 15 fundatrigeniae, measured) and alatae (15 measured) were collected between 11th April and 4th May, 2010 in “Juan Carlos I park”, Barajas (Madrid, Spain) [40°28'12.77"N, 3°35'6.22"W] (reference M-222). Several populations were also located on the same host on 7th May, 2010 in Torrejón de Ardoz (Madrid, Spain) [40°27'23.17"N, 3°28'3.02"W] (M-224) and at the “Vallecas Villa” railway station (Madrid, Spain) [40°22"6.23"N, 3°37'1.15"W] (M-225) on 17th May, 2010. These samples are deposited in the aphid collection of the University of León, Spain and the samples of associated ants in the Collection of the Universidad Autónoma de Barcelona, Spain (Dr. Xavier Espadaler).

## Description of the forms of Schizaphis piricola on primary host

**Figure 2.**
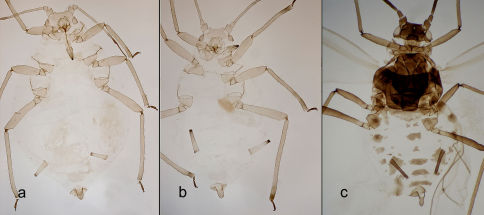
Fundatrix (**A**), apterous fundatrigenia (**B**) and alate fundatrigenia (**C**).

**Figure 3.**
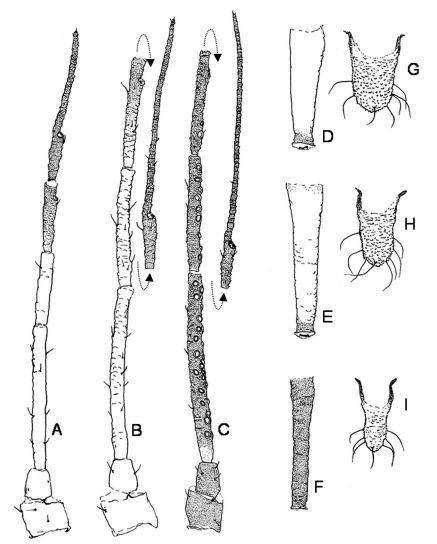
Antennae (**A, B, C**), siphunculi (**D, E, F**) and cauda (**G, H, I**) of fundatrix (**A, D, G**), apterous fundatrigenia (**B, E, H**) and alate fundatrigenia (**C, F, I**) of Schizaphis piricola (Matsumura).

The apterous fundatrigeniae (Figs 1C, 2B, 3B, E, H) are between 1.47 and 2.50 mm long and yellowish green to green, with pale antennae and siphunculi bearing dark tips and dark tarsi. Antennae 0.65 to 0.90 times the body; processus terminalis of antennal segment VI 3.13 to 3.92 times its base, and 0.97 to 1.13 times antennal segment III. Antennae without secondary rhinaria. Apical rostral segment 0.90 to 1.09 times second segment of posterior tarsi and usually with 2 accessory setae. Dorsal sclerotization absent. Marginal papillae on abdominal segments I to VII, absent on III to V in some specimens. Dorsoabdominal setae of anterior terguites shorter (17 to 30 μm) than those of posterior (55 to 90 μm), ventral setae intermediate in size (45 to 60 μm). Siphunculi cylindrical, with weakly defined subapical constriction, 0.12 to 0.19 times body and 1.84 to 2.12 times cauda. Cauda 0.17 to 0.19 mm, 1.21 to 1.46 times basal width, bearing 7 to 9 setae. Tibiae of posterior legs 0.44 to 0.50 times body.

The fundatrices (Figs 2A, 3A, D, G) resemble the apterous fundatrigeniae except for the characteristics of the “fundatrix facies” (Lees 1961; Miyazaki 1987): shorter antennae (1.05–1.25 mm and 0.40 to 0.47 times body), processus terminalis of antennal segment VI (1.82 to 2.18 times base and 0.71 to 0.72 times antennal segment III), siphunculi (approximately 0.10 times body and 1.47 to 1.53 times cauda) and legs (posterior tibiae 0.35 to 0.37 times body).

Alatae fundatrigeniae (Figs 1D, 2C, 3C, F, I) 2.10 to 2.52 mm, green, antennae and siphunculi dark, cauda lighter in colour. Well-pigmented marginal sclerites in terguites II to IV, postsiphuncular sclerites fully developed and spinopleural pigmented bands always present on abdominal segments VI to VIII and also occasionally on III to V. Antennae 0.71 to 0.80 times body; processus terminalis of antennal segment VI 3.41 to 5.18 times base; antennal segments III, IV and V bearing 15–32, 7–18, 0–6 secondary rhinaria, respectively. Siphunculi 1.68 to 1.80 times cauda. The remaining characters are similar to those of the apterae.

## Distribution

Schizaphis piricola (Matsumura) is an aphid of oriental origin which, according to Holman (2009), had only been recorded in China (north east, south east and Taiwan), Japan and Korea, though Lee et al. (2002) have records for India and Pakistan.

Nevertheless, it is possible that Schizaphis piricola is now more widely distributed because Pyrus calleryana is a very commonly planted ornamental tree species. For example, in the United States there is evidence that this tree species is rapidly becoming invasive in much of its horticultural range (Vincent 2005) and it is possible that the aphid is present at this moment.

## Biology

It is a holocyclic dioecious species with species of pear tree (Pyrus sp.) as its primary host and Cyperaceae (Carex spp. and Cyperus rotundus L.) as secondary host (Miyazaki 1988; Blackman and Eastop 1994; Eastop and Blackman 2005; Blackman and Eastop 2006). It has been recorded on Pyrus x* bretschneideri* Rehder, Pyrus communis L., Pyrus pyrifolia (Burm. Fil.) Nakai and Pyrus ursuriensis Maxim. (Blackman and Eastop 1994; Holman 2009). There are also records on Prunus persica (L.) Batsch in Japan (Higuchi and Miyazaki 1969; Moritsu 1983) which we believe should be confirmed. Based on all of these data, this is the first record of an aphid species on Pyrus calleryana (Fig. 1).

In spring in Spain, colonies of this species cause the leaves of Pyrus calleryana to curl (Fig. 1B) as occurs in Pyrus pyrifolia in Japan, Korea and China (Essig and Kuwana 1918), and are attended by the ant Tapinoma nigerrimum (Nylander). The fundatrices appear at the beginning of April and the alatae leave the primary host in mid June to colonize their secondary hosts. Efforts to locate the virginogeniae in these hosts in summer, or alatae re-migrating to the primary hosts in autumn, have so far produced no results.

## Damage to the host plant

The direct action of sucking by the aphids (clearly seen in the curling of the leaves), and indirect damage caused by the honeydew they excrete, which covers the leaves, can affect the normal growth of the trees, all the more so if other aphid species (Aphis pomi De Geer 1773 and Dysaphis sp.) sometimes forming mixed colonies with Schizaphis piricola, are present.

The trees of Pyrus calleryana in “Juan Carlos I park” (Barajas, Madrid, Spain), which have been monitored more carefully, were planted two years ago and do not seem to have reached the height and size expected for this species. In any case, a more in-depth study of the population dynamics and auxiliary fauna (coccinellidae, syrphids, etc…) is necessary, taking into account other variables (humidity, temperature, etc…), to be able to reach conclusions on possible damage.

## Identification keys

The following keys enable all the species in the subgenus Schizaphis recorded in the Iberian Peninsula to be separated.

### Species key (apterae viviparae females)

**Table:** 

1	Siphunculi pale with pigmented apex (Figs 2A, B, 3D, E)	2
–	Siphunculi entirely dark	4
2	Siphunculi 0.8 times cauda at most. Usually on Phalaris arundinacea	Schizaphis (Schizaphis) longicaudata
–	Siphunculi at least 0.9 times cauda. On many species of Gramineae and on Pyrus calleryana	3
3	Siphunculi 1.1 to 1.6 times cauda. On many species of Gramineae	Schizaphis (Schizaphis) graminum
–	Siphunculi 1.82 to 2.1 times cauda in apterous fundatrigeniae (Figs 2B, 3E) and 1.47–1.53 in fundatrices (Figs 2A, 3D). On Pyrus calleryana (primary host)	Schizaphis (Schizaphis) piricola
4	Processus terminalis of antennal segment VI 3.6 to 4.5 times its base. Siphunculi 2.5 times cauda at most. Marginal papillae on abdominal segments I, VI and VII. On Pyrus communis (primary host) or Ciperaceae (secondary host)	Schizaphis (Schizaphis) pyri
–	Processus terminalis of antennal segment VI 4.7 to 6.8 times its base. Siphunculi at least 2.5 times cauda. Marginal papillae on abdominal segments I and VII only. On Cyperus and seldom other plants	Schizaphis (Schizaphis) rotundiventris

### Species key (alate viviparous females)

**Table:** 

1	Siphunculi pale with pigmented apex	2
–	Siphunculi entirely dark (Figs 2C, 3F)	3
2	Siphunculi 0.8 times cauda at most. Normally on Phalaris arundinacea	Schizaphis (Schizaphis) longicaudata
–	Siphunculi at least 0.9 times cauda. On many species of Gramineae and on Pyrus calleryana	3
3	Siphunculi same size as cauda. Bearing 4–10, 0–4 and 0–1 secondary rhynaria on antennal segments III, IV and V, respectively. On many species of Gramineae	Schizaphis (Schizaphis) graminum
–	Siphunculi (Figs 2C, 3F) 1.6 to 1.8 times cauda. Bearing 18 to 32, 7–18 and 2–6 secondary rhynaria on antennal segments III, IV and V, respectively. On Pyrus calleryana (primary host)	Schizaphis (Schizaphis) piricola
4	Siphunculi 0.1 times body at most and 1.5 to 1.8 times cauda. Processus terminalis of antennal segment VI 3.6 to 5.0 times its base and 1.1 to 1.6 times antennal segment III, and approximately 2.0 times siphunculi. On Pyrus communis (primary host) and Ciperaceae (primary host)	Schizaphis (Schizaphis) pyri
–	Siphunculi at least 0.1 times body and 1.1 to 3.0 times cauda. Processus terminalis of antennal segment VI 4.7 to 6.0 times its base and 1.3–1.8 times antennal segment III. On Cyperus	Schizaphis (Schizaphis) rotundiventris
